# Protective effect of MP-40 mitigates BDL-induced hepatic fibrosis by inhibiting the NLRP3-mediated pyroptosis

**DOI:** 10.3389/fphar.2024.1479503

**Published:** 2024-09-20

**Authors:** Xuedong Wan, Yuanyuan Fang, Minjing Qin, Qitong Zheng, Qiao Yang, Mengyun Peng, Min Hao, Kuilong Wang, Ruihua Zhao, Yiqing Shi, Xin Han, Xia’nan Sang, Gang Cao

**Affiliations:** ^1^ School of Pharmacy, Zhejiang Chinese Medical University, Hangzhou, China; ^2^ Songyang Institute, Zhejiang Chinese Medical University, Lishui, China

**Keywords:** Radix Paeoniae Alba, MP-40, bile duct ligation, hepatic fibrosis, NLRP3

## Abstract

**Background:**

Hepatic fibrosis and its associated consequences continue to pose a substantial global health challenge. Developing novel approaches to hepatic fibrosis management and prevention is critically necessary. Radix Paeoniae Alba (RPA) is widely used in Traditional Chinese Medicine (TCM) to treat various diseases. Our earlier research found that a bioactive component of RPA had a dose-dependent effect on anti-allergic asthma. RPA reduces allergic asthma by slowing the hepatic wind, according to “Treatise on Febrile Diseases”. However, this bioactive fraction’s pharmacological effects and mechanisms on the liver are unknown.

**Aim:**

This study examined the bioactive fraction MP-40, the methanol extract of RPA (MRPA), on bile duct ligation (BDL) for its anti-hepatic fibrosis activity and potential mechanisms.

**Methods:**

First, the effectiveness of MP-40 in treating BDL-induced hepatic fibrosis in mice and rats was evaluated through survival rates, ALT, AST HYP, and pathological changes. Molecular assays were performed using *in vitro* cultures of HSC-T6 activation. The expression of α-SMA and Collagen I evaluated fibro-tropic factors with HSC activation. Furthermore, the levels of pyroptosis were assessed by examining the expression of the pyroptosis-related proteins, including NLRP3, Cleaved Caspase-1, GSDMD-N, and 1L-1β. Additionally, the effective constituents of MP-40 were identified by extraction, separation, and identification. Finally, PF and TGG, as the delegate compounds of MP-40, were tested to confirm their inhibition effects on HSC-T6 activation.

**Results:**

The findings demonstrated that MP-40 and MRPA could lower ALT, AST, and HYP levels, boost survival rates, and reduce liver damage in BDL mice and rats. Furthermore, MP-40 outperforms MRPA. MP-40 was proven to drastically diminish fibrotic α-SMA and Collagen I. The expression of pyroptosis-related proteins NLRP3, Cleaved Caspase-1, TGF-β1, GSDMD-N, and 1L-1β decreased. MP-40 inhibited the synthesis of pyroptosis-related proteins more effectively than MCC950 (an NLRP3-specific inhibitor). Monoterpene glycosides and tannins were shown to be the most potent MP-40 components. Finally, the delegate compounds MP-40, PF, and TGG were shown to have substantial inhibitory effects on HSC-T6 activation.

**Conclusion:**

The results proved that MP-40 alleviates BDL-induced cholestatic hepatic fibrosis by inhibiting NLRP3-mediated pyroptosis. PF and TGG play a role in treating BDL-induced cholestatic hepatic fibrosis in MP-40.

## Graphical Abstract

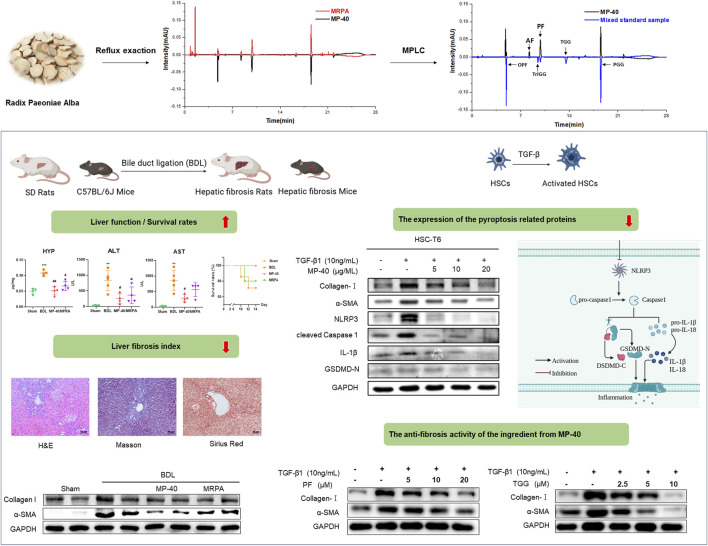

## 1 Introduction

Cholestatic liver disease is a disease that causes liver damage and fibrosis owing to bile stasis. Bile acids constitute the primary components of bile, and an excessive accumulation in the liver can damage the liver and bile ducts. If not timely intervention, it will gradually develop hepatic fibrosis and eventually lead to liver failure. At present, Ursodeoxycholic acid is the most used drug to treat cholestatic liver disease in the clinic. However, some patients do not respond to this treatment (Wagner and Fickert, 2020). Therefore, there is an urgent need to find new and effective drugs to treat these cholestatic liver diseases (Zhao et al., 2015).

As medical research advances, Traditional Chinese medicine (TCM) is gradually becoming one of the popular areas of study. Radix Paeoniae Alba (RPA) is the dried root of *Paeonia lactiflora* Pall. It is usually used to treat diseases related to the liver in the field of TCM. In our previous studies, FB-40, the most bioactive fraction of stir-fried RPA, significantly affects anti-allergic asthma in dose dependence (Sang et al., 2022; Sang et al., 2023). The ancient Chinese medical book “Treatise on Febrile Diseases” refers to RPA relieving allergic asthma by calming the liver wind. Pulmonary dysfunction can occur in cirrhosis of the liver incline. For example, Hepatopulmonar syndrome (HPS) is one of the pulmonary disorders unique to chronic liver diseases. Rat HPS develops in BDL-induced, not thioacetamide (TAA)-induced or CCl_4_-induced cirrhosis (Chen et al., 2021; Qian et al., 2023). BDL is a classical model related to hepatic fibrosis induced by cholestasis, which causes persistent bile acid accumulation through the ligation of bile ducts and leads to chronic inflammation in the liver. BDL has the characteristics of rapid modeling, high repeatability, and prominent hepatic fibrosis (Zhao et al., 2021). According to previous research, HSCs are the primary source of myofibroblasts, which secrete excessive extracellular matrix to exacerbate hepatic fibrosis. Its degree of activation is an essential and widely accepted indicator of assessing the severity of hepatic fibrosis. However, studies have demonstrated that RPA significantly inhibits hepatic fibrosis induced by TAA and CCl_4_ (Zhao et al., 2013), and no relevant research has been conducted currently investigating the effects of RPA on bile duct ligation-induced hepatic fibrosis (Zhao et al., 2013).

This experiment will use the model of BDL in mice and rats to investigate the therapeutic effect of MRPA and its essence part MP-40 on cholestasis-induced hepatic fibrosis. Estimate the impact of RPA on BDL-induced hepatic fibrosis through serum biochemistry and liver histopathology. Finally, the cytotoxicity and anti-HSC activation of Paeoniflorin (PF) and 1,2,3,6-Tetragalloylglucose (TGG), which were the delegate compounds of MP-40, were detected to explore their potential for treating hepatic fibrosis.

## 2 Materials and methods

### 2.1 Materials and reagents

RPA was acquired from Zhejiang University of Chinese Medicine Chinese Herbal Pieces Co., Ltd. PRA (2 kg) was extracted with methanol (1:10 w/v) by reflux exaction three times for 2 h each time. The combined extracts were concentrated and referred to as MRPA (272 g, 13.6%, w/w). 136 g MRPA using Medium Pressure Liquid Chromatography (MPLC, ODS eluting) with MeOH-H_2_O (20:80, 40:60 v/v) to give two subfractions. After decompressing and concentrating the 40:60 v/v MeOH-H_2_O fraction from MRPA, the extract concretes MP-40 (34.5 g, 25.3% to MRPA, w/w) was obtained.

### 2.2 The component analysis of each fraction from MP-40 and MRPA by UPLC

UPLC would analyze the samples of MP-40 and MRPA. The elution conditions of each fraction were as follows: ACQUITY UPLC®BEH C_18_ column (2.1 mm × 50 mm, 1.7 μm); A contained 0.1% formic acid water, while B contained acetonitrile; the injection volume was 1 μL, and the flow rate was 0.3 mL/min. 30°C is the column temperature; Gradient elution: 0→1.2 min, 95% A; 1.2→1.6 min, 95%→89% A; 1.6→4 min, 89% A; 4→8 min, 89%→82% A; 8→9 min, 82%→81% A; 9→14 min, 81% A; 14→17 min, 81%→5% A; 17→18 min, 5% A; 18→19 min, 5%→95% A; 19→24 min, 95% A.

### 2.3 Establishment and drug treatment of BDL-induced hepatic fibrosis

**FIGURE 1:**
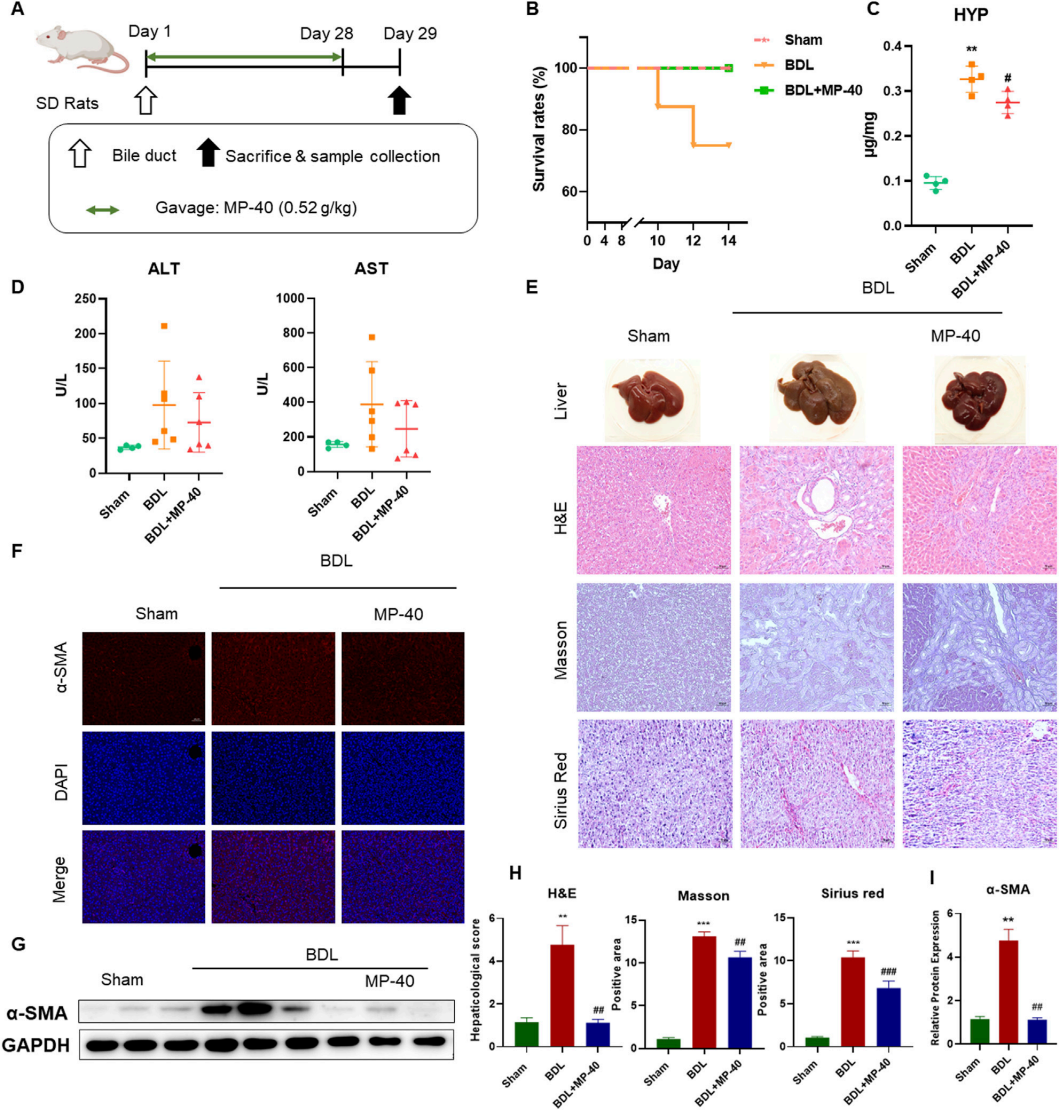
MP-40 prevents hepatic fibrosis in the BDL rat model. **(A)** The experimental procedure of rats. **(B)** Mortality rates. **(C)** HYP indicators in the liver. **(D)** Serum ALT and AST activities. **(E)** Liver appearance pictures, H&E staining, Masson staining, and Sirius Red staining present in × 200 magnification. **(F)** Immunofluorescence staining of α-SMA. **(G)** The bands of α-SMA and GAPDH. **(H)** The histopathological scores and positive areas were quantified. **(I)** The Western blotting analysis of the protein expression of α-SMA in the BDL rat model. **P < 0.01, ***P < 0.001 vs. sham group, ^##^ *P* < 0.01, ^###^ *P* < 0.001 vs. BDL group, ns, not significant.

**FIGURE 2:**
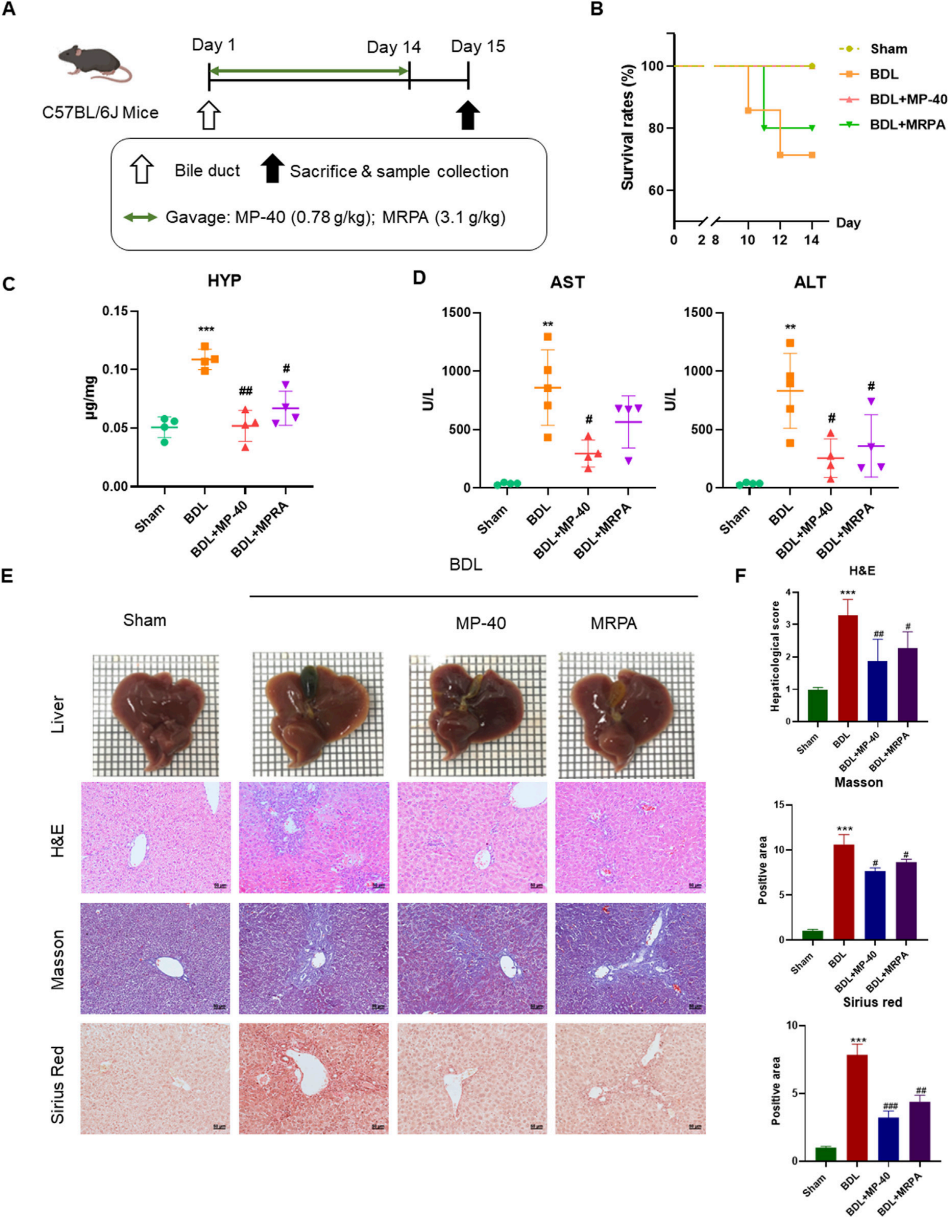
MP-40 and MRPA prevent hepatic fibrosis in the BDL mouse model. **(A)** The experimental procedure of mice. **(B)** Mortality rates. **(C)** HYP indicators in the liver. **(D)** Serum ALT and AST activities. **(E)** Liver appearance pictures, H&E staining, Masson staining, and Sirius Red staining present in × 200 magnification. **(F)** The histopathological scores and positive areas were quantified. **P < 0.01, ***P < 0.001 vs. sham group, ^#^ *P* < 0.05, ^##^ *P* < 0.01, ^###^ *P* < 0.001 vs. BDL group, ns, not significant.

All animals were purchased from the Laboratory Animal Research Center of Zhejiang Chinese Medicine University. Eight-week-old male C57BL/6J mice (20 ± 1 g) and SD rats (200 ± 10 g) were provided by SLAC Laboratory Animal Co., Ltd. (Shanghai, China). All animals were kept in SPF animal rooms with constant temperature (22°C), 12/12 h light/dark cycle, and given free water and food. All mice were acclimated to the environment for 1 week before the experiment began. Four experimental groups were randomly selected from the twenty-two C57BL/6J mice: a sham group (n = 5), a BDL group (n = 7), a BDL + MP-40 group (n = 5), and a BDL + MRPA group (n = 5). The abdomen was opened by a midline incision of about 2 cm following anesthesia with Zoletil 50. The common bile duct was shown after the duodenum was turned out. Using 6–0 surgical grade-sutures, the common bile duct was carefully divided and doubly ligated at both sites. Finally, the surgical operation was then completed by flushing the abdominal cavity with normal saline and closing the skin and subcutaneous wounds separately. In the sham group, only a laparotomy was performed without bile duct ligation, followed by closure of the abdominal cavity. 24 h after surgery, the mice in the MRPA group and MP-40 group were orally administered with 3.1 g/kg RPA methanol extract or 0.78 g/kg MP-40 for 14 days, whereas volume-matched 0.5% carboxymethylcellulose sodium was administered to the sham and BDL groups. (CMC-Na) (Figure 2A). The dosage of MP-40 was based on our previous research (Sang et al., 2023). Three experimental groups consisting of twenty rats each were randomly assigned: sham group (n = 5), BDL group (n = 8), BDL + MP-40 group (n = 7), and the rats in the MP-40 group were given 0.52 g/kg MP-40 for 28 days. The treatment methods of each group of rats were the same as above (Figure 1A). After fasting for 12 h, all animals were anesthetized with Zoletil 50, and cardiac blood was collected. Serum and liver tissues were collected and stored at −80°C. The ethical committee number for the study is IACUC-20231016-13.

### 2.4 Biochemistry indicators of liver function and collagen content

Aspartate transaminase (AST) and alanine aminotransferase (ALT) biochemistry parameters were measured using the serum samples. Additionally, the liver hydroxyproline (HYP) concentration was found using alkaline hydrolysis to identify the collagen content in liver tissue.

### 2.5 Liver histological analysis

The experimental animals’ livers were removed, and a portion of them was fixed with 10% formaldehyde solution for 1 week to inhibit tissue autolysis and maintain structure, then embedded in paraffin, sectioned in 5 μm thick, and stained with H&E, Masson, and Sirius Red for histopathological examination. Then, the sealed sections were placed in the light microscope under × 200 magnification, the morphology of hepatocytes, and were to analyze the liver’s histology and degree of fibrosis. HE stained sections were used to assess the inflammatory infiltration in each group on a 1–4 scale, respectively presenting none, slight, middle, and severe and recording a 1–4 score. Sirius Red and Masson staining sections evaluated the degree of hepatic fibrosis by assessing the collagen deposition area on a 1–5 scale and recording a 1–5 score, respectively. All sections were evaluated by an expert pathologist blind to the experimental manners.

### 2.6 Western blotting analysis in liver tissue

The liver tissue homogenates were stored in freeze lysis buffer and centrifuged at 12,000 rpm at 4°C for 10 min for protein isolation. Protein samples were separated using sodium dodecyl sulphate-polyacrylamide gel electrophoresis (SDS-PAGE), transferred to a PVDF membrane, sealed for 1 h with 5% skim milk, and then incubated for an entire night at 4°C with a particular primary antibody, including NLRP3 (cs5101), α-SMA (ab7871), Collagen I (BS-0578R), Caspase-1 (cs24232), TGF-β1 (ab179695), GAPDH (6000-1-1g), IL-1β (16806-1-AP), and GSDMD (AF4012). Following four PBST washes, the sample was incubated for an hour at room temperature with a 1:5,000 dilution of HRP-conjugated secondary antibody. Ultimately, Clarity Western ECL (Beyotime Biotechnology, Shanghai, CN) densitometry in the membrane was examined using ImageJ.

### 2.7 Immunofluorescence staining in liver tissue

The liver tissue was wrapped using the optimal cutting compound (OCT), and the slices had a 4 μm thickness. Slices were kept in storage at −80°C. After cutting the liver tissue, it was allowed to sit at room temperature for 15 min. Then, it was fixed for 10 min using a fixing solution (methanol: acetone = 1:1). Following a PBS wash, it was sealed for 1 h with 5% sheep serum and left to incubate with the primary antibody at 4°C for the entire night. Secondary antibodies (Goat anti-rabbit IgG H&L Alexa-Fluor^®^ 488 (ab150077); Goat anti-Mouse IgG H&L Alexa-Fluor^®^ 647 (ab150115); Abcam, Cambridge, MA, United States) were put in the sections and given a one-hour dark incubation period at room temperature. After washing, the tablets were examined under a fluorescence microscope and sealed with anti-fluorescence quenching tablets containing 4′,6-diamidino-2-pjemylindole (DAPI).

### 2.8 Cell culture

10% fetal bovine serum (FBS) was added to HSC-T6 cells, which were grown in a humidified atmosphere with 5% CO_2_ at 37°C (Gibco, Carlsbad, CA, United States) and antibiotics (100 μg/mL streptomycin and 100 μg/mL penicillin). When the confluence of cells reached 70%–80%, HSC-T6 cells were inoculated into 96-well plates at a density of 1 × 10^5^ per well and incubated for 24 h. The PF, TGG, and MP-40 were added into the precultured HSC-T6 cell, respectively, and the concentration of the addition double gradient was diluted from 100 μM to 0.78 μM. Following a 24-h incubation period, an MTT assay was conducted to evaluate the toxicity of every monomer towards HSC-T6. HSC-T6 cells have been grown at a density of 1 × 10^6^ per well in 6-well plates following stimulation with 10 ng/mL TGF-β1. They were then treated for 24 h with PF (5, 10, 20 μM), TGG (2.5, 5, 10 μM), and MP-40 (5, 10, 20 μg/mL). After the cells were gathered, a Western blot was performed.

### 2.9 Statistical analysis

GraphPad Prism 8.0.2 (GraphPad InStat Software, San Diego, California, United States) examined all of the experiment data. One-way analysis of variance (One-way ANOVA) was used to compare each group. The mean ± Standard Deviation (SD) was used to express the experiment data. Furthermore, there is statistical significance if *P* < 0.05.

## 3 Results

### 3.1 Survival rates in the BDL rats

The livers of the MP-40-treated rats looked healthy and comparable to those of the sham-operated group, in contrast to the BDL rats (Figure 1E). Rat survival rates in the BDL group (7/7, 100%) were significantly higher than those in the BDL group (6/8, 75.0%) because of this finding (Figure 1B).

### 3.2 MP-40 prevents hepatic injury and hepatic fibrosis in the BDL rats

Compared with the Sham group, the serum ALT and AST levels were elevated in the BDL group. MP-40 (0.52 g/kg) administrations decreased serum AST and AST levels compared with the BDL group, while the activity of ALT and AST had no statistically significant differences. Because the survival rate of mice in the late stage of liver fibrosis was increased by MP-40, the AST and ALT values in the MP-40 group contained data indicating the severity of fibrosis, so there was no significant difference in AST and ALT. HYP activity was higher in the BDL group than in the sham group, suggesting that the cholestasis modeling was successful. The MP-40 therapy also caused a decrease in HYP activity. (The fibrosis index determination results showed a decreasing trend in the MP-40 group but a signal increase in the HYP levels in the BDL group) (Figures 1C, D) BDL animals showed parenchymal necrosis and many newly created bile ducts in liver appearance images and H&E. At the same time, MP-40 could lessen enhanced BDL-induced histological alterations (Figure 1E). Likewise, immunofluorescence staining. Western blotting Analysis indicated that BDL significantly increased α-SMA protein expressions compared with the Sham group, and MP-40 administrations ameliorated these changes caused by BDL (Figures 1F–I).

### 3.3 Survival rates in BDL mice

The livers of BDL mice showed a harsh cirrhotic look on their surface. On the other hand, the liver surface of the anti-MP-40-treated animals seemed shiny and smooth, much like the group that did not have a sham operation (Figure 2A). MP-40 can improve the survival rates of BDL mice (Figure 2B) (survival number: BDL (5/7, 71.4%), MP-40 (5/5, 100%), MRPA (4/5, 80.0%)).

### 3.4 Effect of MP-40 and MRPA on biochemistry indicators of liver function in BDL mice

The serum AST and ALT are the indexes of hepatic cell damage, while hydroxyproline is considered a marker of hepatic fibrosis. The serum levels of AST and ALT were significantly elevated in the mice of the BDL group, showing severe hepatotoxicity. Compared with serum markers of BDL groups, the administration of MRPA or MP-40 significantly decreased the serum levels of ALT, AST, and HYP, respectively (Figures 2C, D), which means that MP-40 and MRPA can alleviate the liver cell injury caused by BDL, and MP-40 took more effect than MRPA.

### 3.5 MP-40 and MRPA relieved BDL-induced histopathological changes in mice

All sections were evaluated by an expert pathologist blind to the experimental manners. The sham mice’s liver imaging revealed a notably smooth surface, a soft texture, and crisp edges. In contrast, the liver of animals with BDL-induced liver disease displayed diffuse nodules with dark red surfaces and various sizes. Around the nodules, the liver tissue was of a darker color and a more intricate texture. The edges were blunt, and the particles were transparent. With MP-40 or MRPA treatment, the particles became diffuser, liver textures became softer, and nodules decreased. The Sham group’s liver morphology with H&E staining was expected, with intact hepatocytes and portal tracts. In the BDL group, infiltration of inflammatory cells in several areas, hepatocyte necrosis, and degeneration were observed. After MRPA or MP-40 treatment, the above pathological injuries were markedly alleviated than those in the BDL group (Figure 2E). The blinded assessment showed that the MP-40 and MRPA groups had significantly lower scores for hepaticological scores than the BDL group. Collagen synthesis, a sign of hepatic fibrosis, was observed using Masson’s trichrome and Sirius red staining. In the BDL group, fibrillary collagen deposition was noted. However, collagen accumulation was strongly attenuated after MRPA or MP-40 treatment. While MP-40 or MRPA showed significantly lower values in the BDL group than in the sham group, positive regions of Masson and Sirius red were more significant in the BDL group than in the sham group. The mouse in the MP-40 group showed better than the MRPA group in improving hepatic fibrosis (Figure 2F).

### 3.6 MP-40 and MRPA attenuate expressions of fibrotropic factors with HSC activation in mice

**FIGURE 3:**
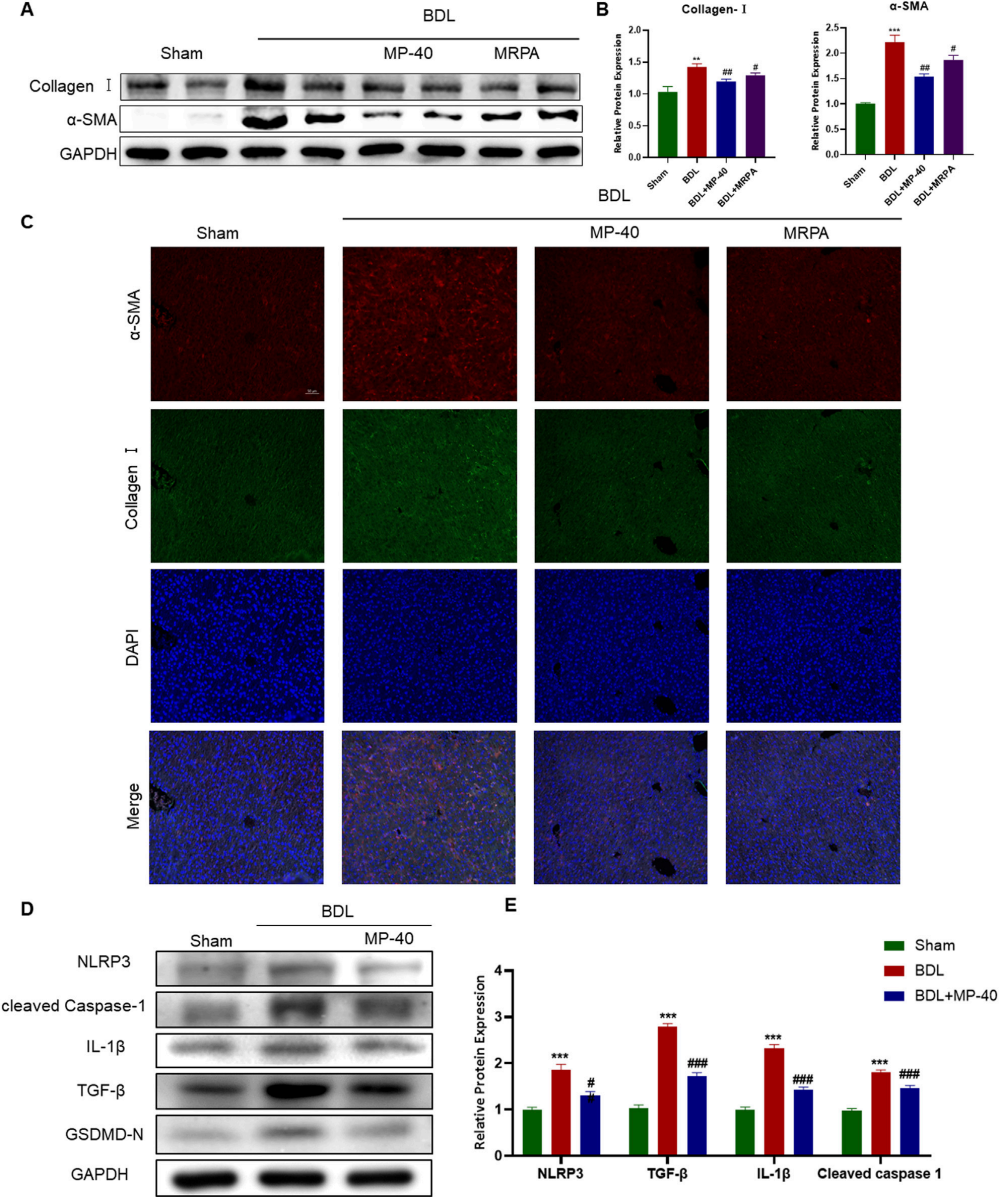
MP-40 and MRPA attenuate expressions of Collagen I, α-SMA, and pyroptosis-related proteins in BDL-induced hepatic fibrosis mice. **(A)** The bands of Collagen I, α-SMA, and GAPDH. **(B)** The Western blotting analysis of the protein expression of Collagen I and α-SMA. **(C)** Immunofluorescence staining of Collagen I and α-SMA. **(D)** The bands of the pyroptosis-related protein expression of NLRP3, Cleaved Caspase-1, IL-1β, and TGF-β1. **(E)** The Western blotting analysis of the protein expression of the pyroptosis-related protein expression by Western blot. *Compared with sham group, **P < 0.01, ***P < 0.001; ^#^Compared with BDL group ^#^ *P* < 0.05, ^##^ *P* < 0.01.

Collagen I and α-SMA levels revealed the degree of HSC activation. Collagen I and α-SMA protein expressions significantly rose in the BDL group vs. the Sham group. After being treated with MRPA or MP-40, the expression of α-SMA and collagen I significantly decreased compared with the BDL group (Figures 3A, B). Using immunofluorescence staining, it was evident that the BDL group, in contrast to the Sham group, had enhanced the immunofluorescence expression of collagen I and α-SMA. Treatment with MRPA or MP-40 decreased the expression of α-SMA compared with the BDL group (Figure 3C), which meant that the two extracts had a significant effect of inhibiting HSC activation induced by BDL, and MP-40 showed a more substantial inhibitory effect than MRPA.

### 3.7 MP-40 mitigates BDL-induced hepatic fibrosis by inhibiting NLRP3-mediated pyroptosis

We identified the NLRP3/Cleaved Caspase-1/IL-1β signaling in the liver to evaluate the impact of MP-40 on hepatic fibrosis and to clarify the mechanism of action of MP-40 in treating hepatic fibrosis. As shown in Figures 3D, E, the protein levels of NLRP3, Cleaved Caspase-1, IL-1β, and TGF-β1 in the liver of the BDL group showed a dramatic increase compared to the Sham group. Moreover, we found that the protein levels of NLRP3, Cleaved Caspase-1, IL-1β, GSDMD-N, and TGF-β1 were decreased after MP-40 treatment in mice. These findings imply that MP-40 may suppress the expression of proteins linked to pyroptosis in the liver of mice with hepatic fibrosis brought on by BDL.

### 3.8 MP-40 suppressed HSC activation by inhibiting NLRP3-mediated pyroptosis

**FIGURE 4:**
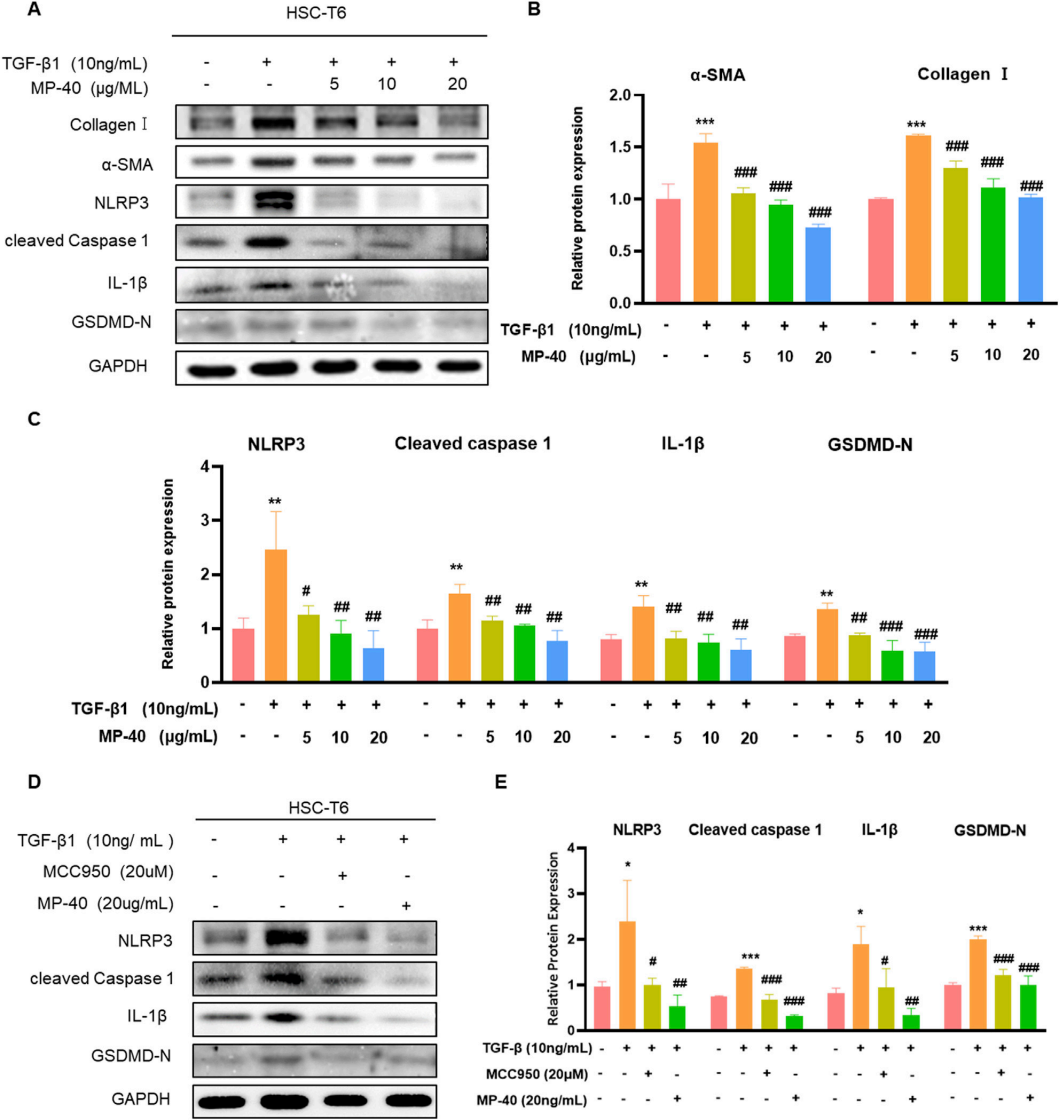
MP-40 reduced the activation of HSC-T6 cells by inhibiting NLRP3-mediated pyroptosis. **(A–C)** Effects of various concentrations of MP-40 on the expression of Collagen I, α-SMA proteins, and pyroptosis-related proteins by Western blot in TGF-β1 treated HSC-T6 cells. **(D, E)** Effect of MCC950 and MP-40 on the NLRP3 signaling pathways in HST-T6 cells after TGF-β1 stimulation. *Compared with Control group, **P < 0.01, ***P < 0.001; ^#^Compared with Model group ^#^ *P* < 0.05, ^##^ *P* < 0.01.

To confirm this *in vitro*, we performed cell experiments. As mentioned in Figures 4A, B, MP-40 treatment decreased the expression of proteins related to hepatic fibrosis, such as α-SMA and collagen I, which suggested that MP-40 suppressed HSC activation. Meanwhile, MP-40 treatment reduced the protein expression of NLRP3, Cleaved Caspase-1, IL-1β, GSDMD-N, and TGF-β1 as the increment of MP-40 dosage (Figures 4A–C). As shown in Figures 4D, E, the expression of related proteins was more strongly reduced by MP-40 than by MCC950 (Mridha et al., 2017; Zhang et al., 2021). These findings suggested that MP-40 exhibited a dose-dependent inhibition of pyroptosis-related protein production and suppression of HSC activation *in vitro*.

### 3.9 Identification of ingredients in MP-40

**FIGURE 5:**
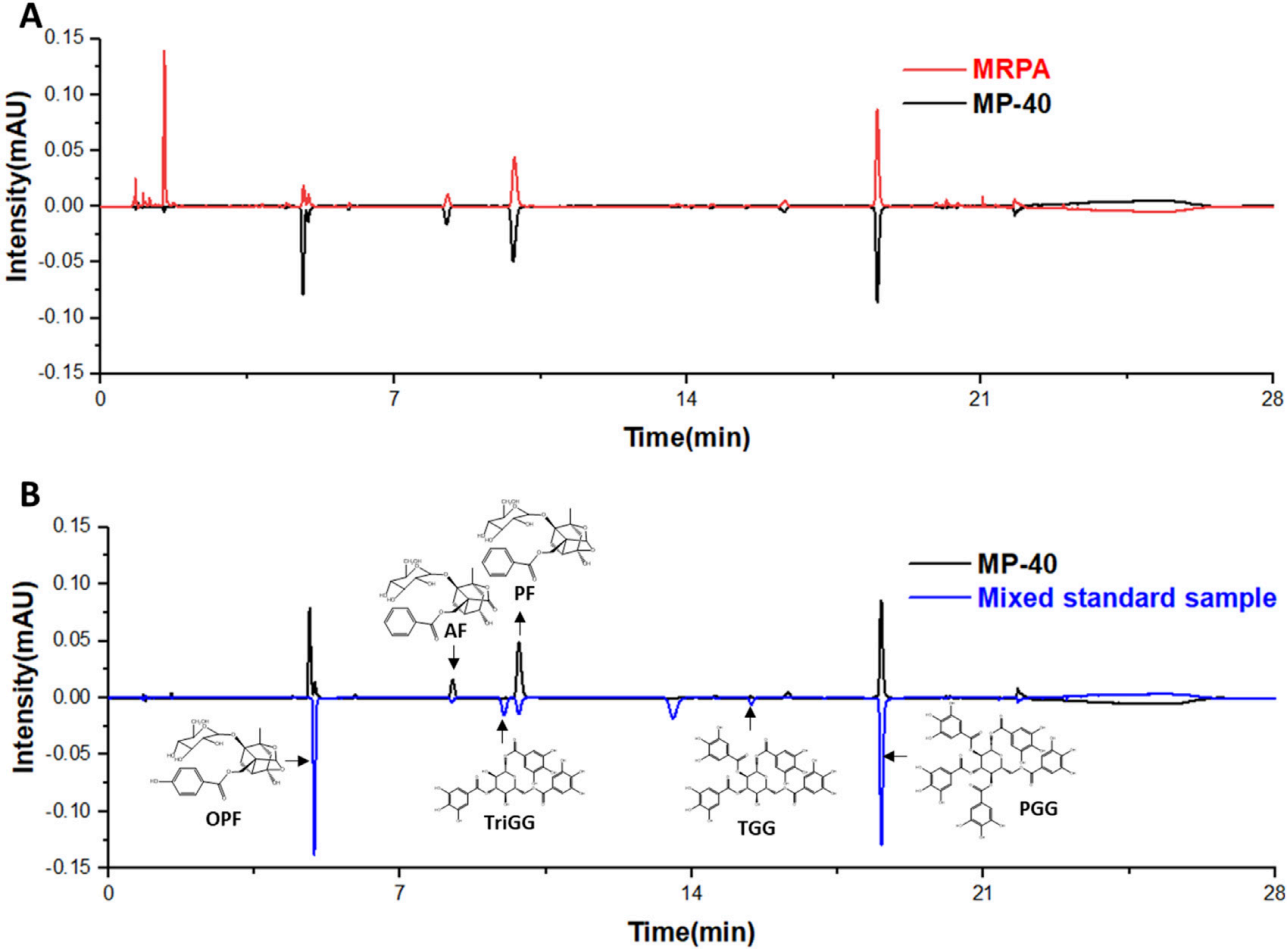
The UPLC results. **(A)** The chromatograms of MP-40 and MRPA. **(B)** The components identification of MP-40.

UPLC analyzed MP-40 and MRPA. The results of the liquid chromatograms of MP-40 and MRPA indicated that MP-40 was a medium-polarity compound of MRPA (Figure 5A). According to comparing with the standard, the main components of MP-40 were Oxypaeoniflorin (OPF), Albiflorin (AF), Paeoniflorin (PF), 1,3,6-Trigalloylglucose (TriGG), 1,2,3,6-Tetragalloylglucose (TGG), and 1,2,3,4,6-O-Pentagalloylglucose (PGG) (Figure 5B).

### 3.10 PF and TGG inhibit the activation of HSC-T6 *in vitro*

**FIGURE 6:**
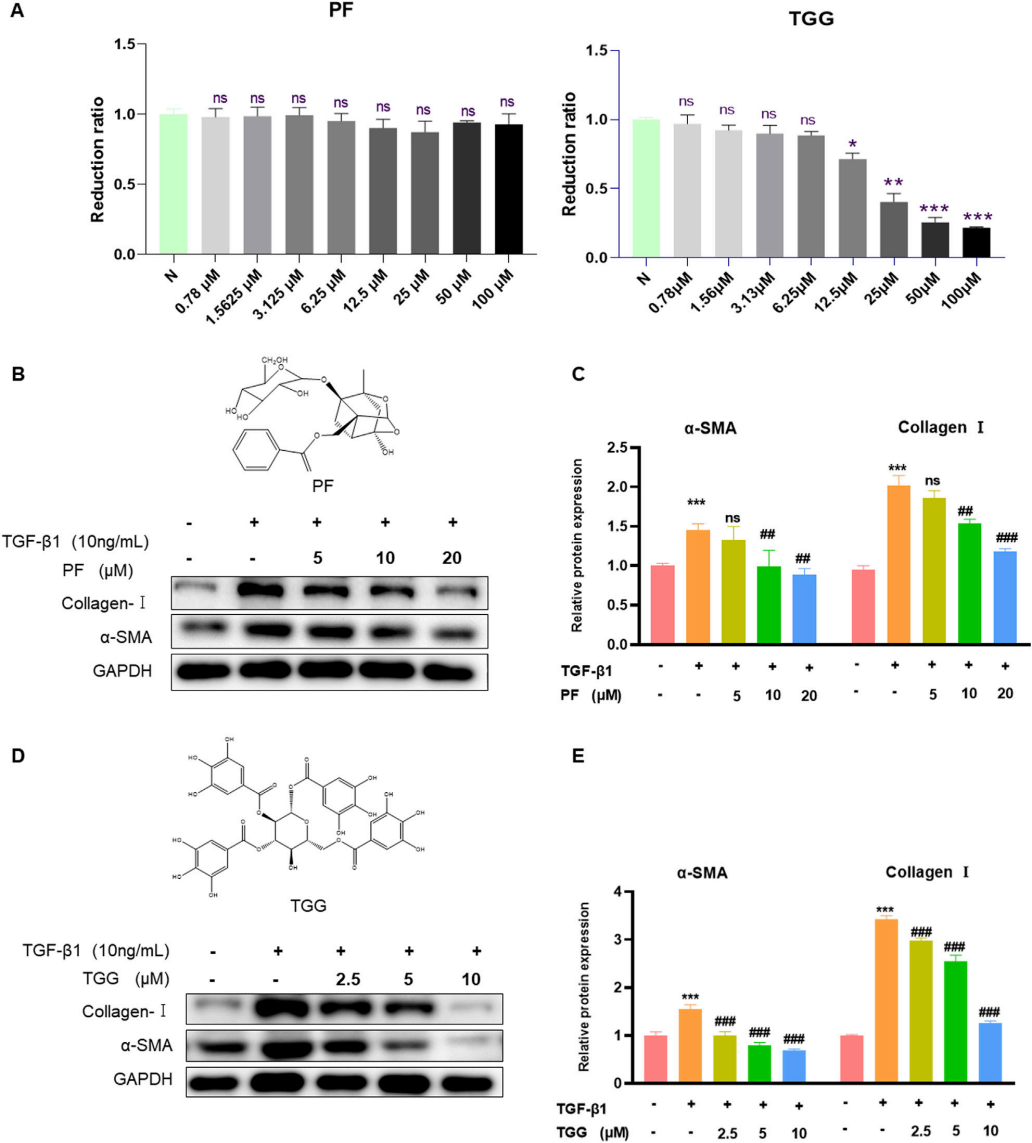
PF and TGG inhibit Collagen I and α-SMA *in vitro*. **(A)** HSC-T6 cells were treated with various concentrations of PF and TGG (0.78–100 μM/mL) for 24 h to assess cell viability. **(B, D)** The bands of Collagen I, α-SMA, and GAPDH. **(C, E)** Representative Western blotting analysis for Collagen I and α-SMA expressions. Densitometric values were normalized against GAPDH. *Compared with Control, *P < 0.05, **P < 0.01, ***P < 0.001; ^#^Compared with TGF-β1 group, ^##^ *P* < 0.01, ^###^ *P* < 0.001. ns, not significant.

To verify the liver protective activity of PF and TGG, the cytotoxic effects were detected in the normal HSC-T6 cells. The outcomes demonstrated that PF respectively performed low cytotoxicity at 100–0.78 μM concentrations. In comparison, TGG showed significantly high cytotoxicity at a concentration of 100–12.5 μM (Figure 6A). Western blotting analysis of α-SMA and collagen-I further demonstrated that HSC activation by TGF-β1 was restrained after treatment. The expression of collagen-I and α-SMA showed that PF and TGG can inhibit the HSC-T6 activation (Figures 6B–E).

## 4 Discussion

In recent years, the incidence of hepatic fibrosis has been increasing, which has caused significant damage to liver tissue, and hepatic fibrosis diseases have gradually attracted widespread attention (Moon et al., 2020; Ding et al., 2023). Due to a variety of acute or chronic liver injuries, hepatic fibrosis is an atypical therapeutic reaction. Principal contributors to hepatic fibrosis were autoimmune diseases, viral hepatitis (hepatitis B; HBV and hepatitis C; HCV), non-alcoholic steatohepatitis, and cholestasis (Friedman, 2008; Zhou et al., 2014). RPA is usually used to treat diseases related to the liver in the field of TCM. In the previous studies, we found that the 40% ethanolic extract from the dichloromethane fraction of stir-fried RPA significantly affects anti-allergic asthma in dose dependence. We separate the 40% ethanolic extract from MRPA to increase the yield. This study found that MP-40 was extracted from MRPA, which has a protective effect against hepatic fibrosis in BDL, which has a significant relationship with lung tissue disease in clinical. In this study, using BDL-induced hepatic fibrosis, we found that MP-40 significantly improved survival rates, downregulated ALT, AST, and HYP, and mitigated damage in the livers of rats and mice. The present work demonstrated that MP-40 significantly decreased the liver’s collagen content and HSC activity. Therefore, MP-40 treats BDL-induced hepatic fibrosis by restraining HSC activity to protect liver function.

It is well documented that the activation of the NLRP3 inflammasome/IL-1β axis can lead to pyroptosis of HSC, thereby exacerbating liver injury and eventually leading to hepatic fibrosis (Kong et al., 2019; Xie et al., 2022). Pro-caspase-1, a key component of NLRP3 inflammasome-mediated pyroptosis, is triggered, resulting in the cleavage of GSDMD (GSDMD-N), which can create holes in the cell membrane. In addition, it stimulates the extracellular release and maturation of IL-1β, a proinflammatory cytokine linked to various pathological conditions. MCC950 (Coll et al., 2015) is an initially identified diaryl sulfonylurea-containing compound that functions as a specific inhibition of NLRP3 (Wu et al., 2020). Further examination of its mechanism revealed that MCC950 hinders the maturation and release of IL-1β via the inhibition of NLRP3 inflammasome activation (Sui et al., 2020). This study found that MP-40 significantly reduced the pyroptosis-related proteins NLRP3, Cleaved Caspase-1, GSDMD-N, and 1L-1β.

The main components of MP-40 were identified by comparison with the standard. After analyzing the main compounds of MP-40, it is found that monoterpene glycosides and tannins are two primary components of MP-40. PF and TGG were used to determine the pharmacological effects on HSC-T6 activation following TGF-β1 treatment *in vitro*. The result showed that PF and TGG effectively inhibited HSC-T6 activation.

In conclusion, the results proved that MP-40 alleviates BDL-induced cholestatic hepatic fibrosis by inhibiting NLRP3-mediated pyroptosis. Consequently, monoterpene glycosides and tannins were identified as the main active ingredients of MP-40 in treating BDL-induced cholestatic hepatic fibrosis as the delegate compounds of monoterpene glycosides and tannins, PF and TGG were confirmed to have significant inhibition effects on HSC-T6 activation. This experiment revealed the potential of the monomer component of MP-40 as a therapeutic drug for anti-hepatic fibrosis and provided a theoretical basis for future research.

## Figures and Tables

**Table:** 

**RPA**	Radix Paeoniae Alba
**MRPA**	Methanol extracts from Radix Paeoniae Alba
**PF**	Paeoniflorin
**TGG**	1,2,3,6-Tetragalloylglucose
**ALT**	Alanine aminotransferase
**AST**	Aspartate aminotransferase
**HYP**	Hydroxyproline
**H&E**	Hematoxylin-eosin staining
**HSC**	Hepatic stellate cells
**α-SMA**	α-smooth muscle actin
**BCA**	Bicinchoninic acid
**SDS-PAGE**	Sodium dodecyl sulfate-polyacrylamide gel electrophoresis
**PVDF**	Polyvinylidene fluoride
**PBS**	Phosphate buffered saline
**PBST**	Phosphate buffered saline with tween-20
**ECL**	Enhanced chemiluminescence
**One-way ANOVA**	One-way analysis of variance
**SD**	Standard deviation
**HIS**	Histamine
**WB**	Western blot
**HBV**	Hepatitis B viral
**HCV**	Hepatitis C viral
**NLRP3**	NOD-, LRR- and pyrin domain-containing protein 3
**IL-1β**	Interleukin-1β
**IL-18**	Interleukin-18
**TGF-β1**	Transforming growth factor-β
**BDL**	Bile duct ligation
**CMC-Na**	Carboxymethylcellulose sodium
**PAI-1**	Plasminogen activator inhibitor-1
**ECM**	Extracellular matrix
**OPF**	Oxypaeoniflorin
**AF**	Albiflorin
**TriGG**	1,3,6-Trigalloylglucose
**PGG**	1,2,3,4,6-O-Pentagalloylglucose
**TCM**	Traditional chinese medicine
**ASC**	Apoptosis associated specle-like protein
**PAMPs**	Pathogen-associated molecular patterns
**DAPMs**	Damage associated molecular patterns
**NF-kB**	Nuclear factor kappa B

## Data Availability

The raw data supporting the conclusions of this article will be made available by the corresponding authors on reasonable request.
